# ICU Admission and Post-Discharge Mortality in COVID-19: Different Risk Factors Across Clinical Phases

**DOI:** 10.3390/medsci14020255

**Published:** 2026-05-14

**Authors:** Fernanda Leite, André Santos Silva, Sara Ferreira, Carina Brito, Ângela Leite

**Affiliations:** 1 Department of Transfusion Medicine, Santo António University Hospital Center, 4099-001 Porto, Portugal; u12565@chporto.min-saude.pt (S.F.); u14890@chporto.min-saude.pt (C.B.); 2 Department of Public Health and Forensic Sciences, and Medical Education, Medical Research Centre Faculty of Medicine, University of Porto, 4200-450 Porto, Portugal; 3 Metabesity-i3S-Institute for Research and Innovation in Health, 4200-135 Porto, Portugal; 4 Department of Infectious Diseases, Santo António University Hospital Center, 4099-001 Porto, Portugal; andresantossilva.infeciologia@chporto.min-saude.pt; 5 Centro de Estudos Filosóficos e Humanísticos, Universidade Católica Portuguesa, 4710-297 Braga, Portugal; aleite@ucp.pt

**Keywords:** COVID-19, patient discharge, hospital mortality, intensive care units, risk factors, obesity, neoplasms, oxygen inhalation therapy, disease progression, follow-up studies

## Abstract

Background: Risk factors for severe COVID-19 and in-hospital mortality are well described, but it remains unclear whether the same factors predict mortality after hospital discharge. Distinguishing risk profiles across clinical phases may improve patient management and follow-up strategies. Methods: We conducted a retrospective observational cohort study of 595 adults hospitalized with PCR-confirmed SARS-CoV-2 infection in Portugal (September–November 2020). The primary outcome was all-cause mortality during hospitalization and up to 120 days post-discharge. Secondary outcomes included intensive care unit (ICU) admission, maximum disease severity (WHO Clinical Progression Scale), oxygen supplementation, and length of stay. Univariable and multivariable regression analyses were performed using logistic regression for binary outcomes and linear regression for continuous outcomes. Results: Overall mortality was 22.5%, rising from 14.1% in-hospital to 22.5% at 120-day follow-up (*p* < 0.001), with 37.3% of deaths occurring post-discharge. ICU admission was required in 17.6% of patients and was significantly associated with obesity (OR = 2.12, 95% CI: 1.39–3.23, *p* < 0.001) and male sex (OR = 1.78, 95% CI: 1.14–2.78, *p* = 0.010) in univariable analysis. In contrast, post-discharge mortality was associated with longer hospital stay (18.4 vs. 9.9 days, *p* < 0.001) and a higher prevalence of malignancy (28.0% vs. 13.1%, *p* = 0.032), but not with ICU admission. In multivariable logistic regression, oxygen supplementation was the strongest predictor of 120-day mortality (OR = 2.50, 95% CI: 1.38–4.51, *p* = 0.002). Only pulmonary diseases and obesity were independently associated with maximum disease severity. Conclusions: Risk factors for acute COVID-19 severity differ from those for post-discharge mortality. These findings support a phase-specific approach to risk stratification, suggesting that patients with obesity are at increased risk of early respiratory deterioration, while patients with malignancy may benefit from closer post-discharge follow-up regardless of ICU admission status.

## 1. Introduction

Coronavirus disease 2019 (COVID-19), caused by severe acute respiratory syndrome coronavirus 2 (SARS-CoV-2), was first identified in late 2019 and rapidly evolved into a global pandemic [1,2]. Early clinical and epidemiological reports described a wide spectrum of disease severity and highlighted the substantial burden of morbidity and mortality associated with COVID-19 worldwide [3,4].

Since the early phases of the pandemic, numerous studies have identified demographic and clinical factors associated with severe disease, intensive care unit (ICU) admission, and in-hospital mortality. Advanced age, male sex, obesity, and multiple comorbidities have consistently been linked to worse in-hospital outcomes across diverse healthcare settings [5,6,7,8]. These findings have informed risk stratification, decision-making, and resource allocation during acute hospitalization.

Several prognostic models and risk scores have subsequently been developed to predict disease severity and mortality in hospitalized patients with COVID-19 [9,10,11]. However, systematic reviews have raised concerns regarding heterogeneity, bias, and limited external validity among many proposed models, underscoring the challenges of prognostic research during a rapidly evolving pandemic [11,12].

As survival following hospitalization for COVID-19 improved, attention increasingly shifted toward outcomes occurring after hospital discharge. Post-acute and long-term sequelae of COVID-19, including persistent symptoms and increased cardiovascular risk, have been increasingly recognized [13,14]. Nevertheless, most studies have focused either on acute in-hospital outcomes or on selected post-acute populations, limiting the ability to directly compare risk factors across different phases of disease within the same cohort.

Pathophysiological studies have demonstrated that severe COVID-19 is characterized by marked inflammatory responses, endothelial injury, and immunothrombotic dysregulation, with biomarkers of inflammation and thrombosis strongly associated with disease severity and poor in-hospital outcomes [15,16,17,18]. However, their relationship with late post-discharge mortality remains less well defined.

In parallel, the widespread implementation of vaccination campaigns and the emergence of new viral variants have altered the epidemiology and clinical course of COVID-19 over time [19,20]. As healthcare systems transition from emergency response toward long-term disease management, there is an increasing need to better characterize phase-specific risk profiles to inform both inpatient care and post-discharge follow-up strategies [21].

Therefore, the aim of the present study was to evaluate risk factors associated with acute disease severity, ICU admission, and mortality during hospitalization and up to 120 days after discharge in a retrospective cohort of patients hospitalized with SARS-CoV-2 infection. By distinguishing predictors of acute versus late outcomes within the same population, this study seeks to inform phase-specific risk assessment across the continuum of COVID-19 care. Importantly, the study enrolment (September–November 2020) and the entire 120-day follow-up period (through March 2021) occurred before the widespread rollout of COVID-19 vaccines in Portugal. Thus, none of the participants had received vaccination, providing a ‘vaccine-naïve’ cohort that reflects the natural history of hospitalized adult patients with SARS-CoV-2 infection unconfounded by vaccine-induced immunity.

## 2. Materials and Methods

### 2.1. Study Design and Patients

We conducted a retrospective observational cohort study of all consecutive adults (≥18 years) hospitalized with PCR-confirmed COVID-19 at the Santo António University Hospital Center, Portugal, during the second wave of the pandemic (1 September–30 November 2020. The study was conducted before the widespread rollout of COVID-19 vaccines in Portugal, and according to national guidelines, individuals with confirmed SARS-CoV-2 infection were required to defer vaccination for six months following infection. Consequently, all patients remained vaccine-naïve throughout the hospitalization and the entire 120-day post-discharge follow-up period. Cases were identified via the hospital’s Epic electronic health records (HER) system. A confirmed COVID-19 case was defined by the presence of ICD-10 code U07.1 and a positive RT-PCR test for SARS-CoV-2 from a respiratory specimen during hospitalization. Inclusion criteria were: (1) age ≥18 years, (2) hospitalization during the study period, and (3) meeting the confirmed COVID-19 case definition. The exclusion criterion was >30% missing clinical data in the HER. Data were sourced from a Portuguese national platform, the National Epidemiological Surveillance System (SINAVE), a mandatory registry of confirmed SARS-CoV-2 infections providing diagnosis dates and hospitalization data. By cross-referencing with SINAVE, we confirmed that no patient had a documented prior infection before the index hospitalization. The study was approved by the hospital’s Scientific Board, Ethics Committee and Data Protection Officer (Reference: 2021.236 [188-DEFI/196-CE]), with anonymized data handling in compliance with data protection legislation and a waiver of informed consent. This study is reported following the Strengthening the Reporting of Observational Studies in Epidemiology (STROBE) [22] guidelines for cohort studies. The completed STROBE checklist is provided as: STROBE checklist Final S1 File.

### 2.2. Baseline Demographic and Clinical Variables

Demographic, clinical, treatment, and outcome data were extracted from HER. Variables included age, sex, comorbidities [obesity (Body Mass Index ≥ 30), pulmonary, cardiac, hypertension, dyslipidemia, type 2 diabetes, renal, oncological, hematological, and other (including auto-immune, neurodegenerative diseases, and conditions in immunosuppression)], symptoms at admission, and admission laboratory values (complete blood count, neutrophil-to-lymphocyte ratio (NLR), renal and liver function tests, CRP, ferritin, fibrinogen, D-dimer). Treatments recorded were dexamethasone, remdesivir, and enoxaparin (categorized as prophylactic or therapeutic). Ventilatory support was categorized from supplemental oxygen to invasive mechanical ventilation. Clinical parameters also obtained included date of hospital admission, hospital admission department and transfers, intensive care unit (ICU) admission date, discharge date, date of death, length of stay (LOS), time between diagnosis and hospitalization, symptoms at admission, complications during hospitalization, and progress after discharge.

### 2.3. WHO Ordinal Scale and Severity Definitions

Maximum COVID-19 severity during hospitalization was assessed using the 9-point WHO Clinical Progression Scale (0 = uninfected; 1–8 = increasing severity of illness, with 8 = death) [23]. For each patient, we recorded the highest (worst) score attained at any point during the hospital stay. This ordinal variable served as a key outcome measure to characterize the peak clinical burden of the disease.

### 2.4. Outcomes

The primary prognostic outcome was all-cause mortality recorded during hospitalization and 120 days after discharge. Secondary prognostic outcomes were LOS (duration of hospitalization in the ICU and medical ward), the need for ventilatory treatment, and the existence of COVID-19 symptoms. Patients still hospitalized at data extraction were excluded from mortality and LOS calculations (Figure S1).

### 2.5. Statistical Analysis

Descriptive statistical analyses were performed to summarize all variables. For categorical data, frequencies and percentages (%) were reported. For continuous variables with normal distribution, means (M) and standard deviations (SD) were calculated, while for non-normally distributed continuous variables, medians (Mdn) and interquartile ranges (IQR) were used. Inferential analyses included bivariate comparisons using Student’s *t*-test for normally distributed continuous variables that met assumptions of normality (Shapiro–Wilk *p* > 0.05) and homogeneity of variance (Levene’s *p* > 0.05), with results reported as t(df), *p*-value, and Cohen’s d for effect size interpretation (cutoffs: 0.2 = small, 0.5 = medium, 0.8 = large). For continuous variables violating parametric assumptions, the Mann–Whitney U test was employed, with reporting of U statistic, z-score, *p*-value, and rank-biserial correlation r effect size (cutoffs: 0.1 = small, 0.3 = medium, 0.5 = large). Categorical variables were compared using Chi-square tests (χ^2^(df), *p*-value) when expected cell frequencies exceeded 5; otherwise, Fisher’s exact test was applied, with Cramér’s V effect size reported (cutoffs: 0.1 = small, 0.3 = medium, 0.5 = large).

Comparison between in-hospital and 120-day mortality was performed using McNemar’s test for paired proportions. Statistical significance was set at *p* < 0.05 for all tests. For binary outcomes (120-day all-cause mortality, oxygen supplementation, symptomatology), multivariable logistic regression was performed, reporting odds ratios (OR) with 95% confidence intervals and Nagelkerke R^2^. Due to the absence of exact death dates for post-discharge deaths, time-to-event analysis was not feasible. Post-discharge mortality was therefore analyzed descriptively (in-hospital vs. post-discharge deaths) using bivariate comparisons, as presented in Section 3.1 and Table 1. For continuous outcomes (length of stay), linear regression was performed, reporting unstandardized (B) and standardized (β) coefficients with 95% confidence intervals, *p*-values, overall model F(df), *p*-value, and adjusted R^2^ after verifying linearity, multicollinearity (VIF < 5 cutoff), and residual assumptions. Moderation analysis tested interaction effects (e.g., ICU transfer moderating oxygen-hospitalization relationship) using Hayes’ PROCESS macro or hierarchical regression, reporting interaction coefficients (B), *p*-values, ΔR^2^ for interaction terms, and simple slopes analysis at ±1 SD of the moderator. Analyses were performed using SPSS Statistics (Version 28.0, IBM Corp., Armonk, NY, USA), with effect sizes reported for clinical interpretation and missing data addressed via appropriate methods (multiple imputation). Missing data across laboratory variables ranged from 0% to 35%, with the highest missingness observed for ferritin (35.0%, *N* = 208) and D-dimer (29.4%, *N* = 175). Complete case analysis and multiple imputation (5 imputations, 10 iterations) yielded comparable results, supporting a missing-at-random assumption. Patients with missing data did not differ significantly from those with complete data in terms of age, sex, or mortality status (*p* > 0.05 for all comparisons). Owing to the large quantity of data generated in this study, we have focused on presenting statistically significant outcomes. Non-significant results are included only when they provide meaningful context for the interpretation of the findings. No additional sensitivity analyses were performed.

## 3. Results

### 3.1. Cohort Demographic and Clinical Characteristics

**Figure 1:**
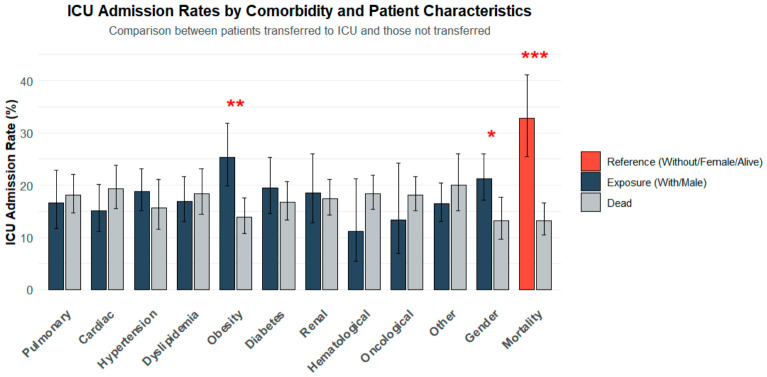
ICU admission rates by comorbidity and patient characteristics. Bars represent the percentage of patients admitted to the intensive care unit (ICU) among those with (dark gray) and without (light gray) each condition. Error bars indicate 95% confidence intervals. Statistically significant differences (*p* < 0.05) between groups are denoted by asterisks (*): * *p* < 0.05, ** *p* < 0.01, *** *p* < 0. 001. Obesity, male sex, and mortality at 120-day follow-up were significantly associated with higher ICU transfer rates, whereas no significant differences were observed for pulmonary, cardiac, hypertensive, dyslipidemic, diabetic, renal, hematological, oncological, or other comorbidities.

Between 1 September and 30 November 2020, a total of 595 adults were admitted to the hospital with SARS-CoV-2 infection or acquired the infection during hospitalization. The sample was predominantly male (55.3%) with a mean age of 70.45 years (SD = 15.26). Most patients (96.1%) were admitted to a medical ward, with 17.6% (*N* = 105) subsequently requiring ICU transfer. ICU admission was significantly associated with obesity (OR: 2.12, 95% CI 1.39–3.23; *p* < 0.001), male sex (OR: 1.78, 95% CI 1.14–2.78; *p* = 0.010), and mortality (OR: 3.21, 95% CI 2.05–5.02; *p* < 0.001) (Figure 1). No significant associations were observed for other comorbidities (Table S1). The mean interval between SARS-CoV-2 infection diagnosis and hospital admission was 2.97 days (SD = 3.82; range, 0–22), and the mean LOS was 12.09 days (SD = 11.50; range, 1–121). Comorbidities were prevalent: hypertension (62.4%), dyslipidemia (48.7%), cardiac pathology (41.0%), type 2 diabetes (34.3%), obesity (32.9%), pulmonary (28.4%); renal (21.8%), hematologic (10.6%) and oncological (other than hematologic) (10.1%). Other comorbidities were present in 65.5% of the population and included autoimmune diseases, neurodegenerative conditions, and immunosuppressive states (including six people living with HIV). Most patients (72.8%) were symptomatic, and 78.2% required some form of oxygen supplementation. Almost a quarter of the studied sample (21.8%) did not need ventilatory support; but about half of the sample (49.2%) received oxygen therapy by cannula; 10.3% required oxygen therapy with a high-output mask, 7.2% underwent invasive mechanical ventilation; 5.9% required continuous positive airway pressure or bilevel positive airway pressure, and, finally, 5.5% received oxygen therapy through high-flow cannulas. Therapeutic interventions included remdesivir (33.9%), dexamethasone (64.7%), and enoxaparin (89.9%), predominantly in prophylactic doses (68.4%).

By 30 November 2020, 84 patients (14.1%) had died during hospitalization. Mortality increased significantly to 134 patients (22.5%) by March 2021 (120-day follow-up), representing an absolute increase of 8.4% (McNemar’s test, χ^2^ = 48.02, *p* < 0.001). The clinical severity trajectory differed significantly between patients who died during hospitalization (in 2020) and those who died post-discharge (till March 2021). Among patients who died, a significant association between the timing of death and COVID-19 severity distribution was verified (χ^2^ (5) = 43.63, *p* < 0.001). The proportion of patients who reached the maximum score of 8 on the ordinal severity scale was significantly higher in the group that died during hospitalization (77/84, 91.7%) compared to the group that died after discharge (20/50, 40.0%).

Deceased patients were significantly older than survivors (M = 79.00, SD = 11.66 vs. M = 67.96, SD = 15.30; t (279.289) = −8.946; *p* < 0.001; d = −0.76). LOS did not differ significantly between groups (deceased: M = 11.82, SD = 11.57), although the interval between diagnosis and admission was longer among those who died (M = 3.14, SD = 3.77 vs. M = 2.37, SD = 3.94; t (593) = 2.074; *p* = 0.038; d = 0.20).

### 3.2. Group Comparisons

#### 3.2.1. Mortality

Non-survivors presented with statistically significant elevations in neutrophil count, NLR, plasma levels of glucose, urea, alkaline phosphatase (ALP), lactate dehydrogenase (LDH), CRP, and D-dimer, alongside lower hemoglobin values (Table 1). Mortality rates were disproportionately higher among patients with oncological, cardiac, renal, and other comorbidities (Table 1).

The administration of remdesivir or dexamethasone showed no significant association with mortality outcomes in univariate analysis. The clinical profile differed significantly between the cohort administered remdesivir and the counterpart. The remdesivir group was marked by a heightened inflammatory response and cellular injury, while patients in the non-remdesivir group showed significantly higher renal and hepatic impairment, suggesting treatment decisions were influenced by disease severity and comorbidities (Table S2). Indeed, patients who received remdesivir had a significantly higher baseline disease severity on the 8-point ordinal scale. The results indicated a statistically significant difference in the distribution of severity scores between the two groups (U = 32,450, *p* < 0.001). An inspection of the mean ranks suggested that patients who received remdesivir had significantly higher (more severe) scores (Mean Rank = 342.1) compared to those who did not receive the drug (Mean Rank = 264.8). Enoxaparin was administered less frequently to deceased patients (18.8%) than survivors (71.1%) [χ^2^ (1) = 7.652; *p* = 0.006; Φ = −0.113]. However, among recipients, a therapeutic dosing regimen was more frequent in the deceased group (41.7% vs. 18.1%) [χ^2^ (1) = 27.021; *p* < 0.001; Φ = 0.226].

To analyze all-cause mortality (deaths up to 120 days post-discharge), we compared two groups: patients who died during hospitalization and those who died between November 2020 and March 2021 after discharge. The in-hospital mortality group showed a significantly shorter mean time from hospital admission to death in comparison with the post-discharge mortality group (*M* = 9.86, *SD* = 8.05 vs. *M* = 18.41, *SD* = 13.68 days, respectively) [*t* (67.73) = −3.992, *p* < 0.001; Cohen’s *d* = −0.817 (95% CI: −1.181, −0.450)]. Moreover, patients who died during hospitalization presented a lower prevalence of oncological comorbidity (13.1% vs. 28.0%) [χ^2^ (1) = 4.588; *p* = 0.032; Φ = 0.185] in comparison with the post-discharge mortality group.

#### 3.2.2. Oxygen Supplementation

Patients requiring oxygen had significantly higher hemoglobin values, neutrophils count, NLR, plasma level of glucose, LDH, CRP, and ferritin, and lower ALP (Table 2). Pulmonary comorbidities, obesity, and diabetes were more prevalent in this group (Table 2). Oxygen-supplemented patients were more likely to receive remdesivir [χ^2^ (1) = 63.833; *p* < 0.001; Φ = 0.328], dexamethasone [χ^2^ (1) = 217.986; *p* < 0.001; Φ = 0.605], and enoxaparin [χ^2^ (1) = 42.949; *p* < 0.001; Φ = 0.269].

#### 3.2.3. Length of Stay (LOS)

LOS correlated negatively with hemoglobin (r = −0.118; *p* = 0.004) and positively with NLR (r = 0.080; *p* = 0.050) and platelet count (r = 0.091; *p* = 0.027). Hospitalization duration was longer for patients with dyslipidemia [t(592) = −2.904, *p* = 0.004, d = −0.238], obesity [t(281.006) = −2.240, *p* = 0.024, d = −0.224], renal [t(147.697) = −2.620, *p* < 0.001, d = −0.366], hematologic [t(65.041) = −2.595, *p* = 0.006, d = −0.617] and other [t(592) = −2.305, *p* = 0.022, d = −0.199] comorbidities. Patients receiving enoxaparin had longer stays [t (592) = −2.248, *p* = 0.025, d = −0.302], particularly those on a therapeutic regimen (M = 16.49, SD = 15.69) versus prophylactic (M = 11.23, SD = 10.16) [*t* (149.591) = −3.466, *p* < 0.001, d = −0.452].

LOS analysis revealed significant differences by mortality outcome (F = 9.37, *p* < 0.001). Post hoc analysis delineated two distinct patterns: patients who died during the initial hospitalization phase had the shortest length of stay (M = 9.9 days, SD = 8.0), which was not significantly different from survivors (M = 11.8 days, SD = 11.5; *p* = 0.435). In contrast, patients who died by the 120-day follow-up had a markedly prolonged hospitalization (M = 18.4 days, SD = 13.7), which was significantly longer than both survivors (mean difference +6.6 days, *p* < 0.001) and early in-hospital decedents (mean difference +8.6 days, *p* < 0.001).

#### 3.2.4. Symptomatology

Of the 595 patients, 433 (72.8%) were symptomatic at admission. As shown in Table S3, symptomatic patients had significantly higher AST (*p* = 0.024) and CRP (*p* = 0.010), lower total bilirubin (*p* = 0.047), and were more likely to receive oxygen supplementation (*p* < 0.001), dexamethasone (*p* < 0.001), and enoxaparin (*p* = 0.042) compared with asymptomatic patients. Due to the substantial imbalance between groups (72.8% vs. 27.2%) and the exploratory nature of this analysis, multivariable modeling was not performed.

### 3.3. Correlations

The pattern of correlations reinforced key clinical relationships. Mortality showed significant positive correlations with several comorbidities (cardiac, renal, oncological) and biomarkers of inflammation (CRP, NLR), cellular injury (LDH), and organ dysfunction (creatinine, urea). Notably, mortality correlated strongly with the need for oxygen supplementation (ρ = 0.360) and therapeutic enoxaparin regimen (ρ = 0.226), while correlating negatively with enoxaparin intake itself (Table S4). Oxygen supplementation showed significant positive correlations with inflammatory markers, including C-reactive protein (ρ = 0.380, *p* < 0.001), neutrophil-to-lymphocyte ratio (ρ = 0.278, *p* < 0.001), and ferritin (ρ = 0.246, *p* < 0.001). Strong positive correlations were also observed between oxygen requirement and COVID-19 therapies, particularly dexamethasone (ρ = 0.558, *p* < 0.001) and remdesivir (ρ = 0.364, *p* < 0.001), reflecting confounding by indication. Enoxaparin use (ρ = 0.238, *p* < 0.001) and therapeutic dosing regimen (ρ = 0.155, *p* < 0.001) also correlated positively with oxygen supplementation.

**Figure 2:**
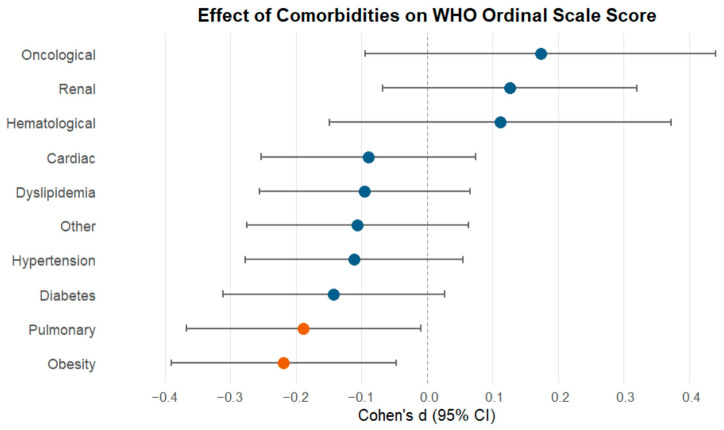
Forest plot of standardized mean differences (Cohen’s *d*) with 95% confidence intervals comparing WHO ordinal scale maximum severity scores between participants with and without each comorbidity. Negative coefficients indicate higher (worse) severity. Pulmonary disease and obesity, marked by orange spots, denote comorbidities independently associated with higher severity (*p* < 0.05).

The strongest positive correlation of disease severity, as measured by the WHO ordinal scale, was with all-cause mortality (ρ = 0.510, *p* < 0.001, *N* = 595). We compared the proportion of patients who reached the maximum score of 8 on the COVID-19 ordinal severity scale between survivors and non-survivors. As expected, none of the 461 survivors had a severity score of 8. In contrast, a substantial majority of non-survivors (97/134, 72.4%) reached this maximum score. The difference in proportions was −72.4 percentage points (95% Confidence Interval [−79.4%, −64.3%] using the Agresti-Caffo method). This difference was highly statistically significant (Wald Z = −18.74, *p* < 0.001). Severity was strongly associated with markers of systemic inflammation, including CRP (ρ = 0.384, *p* < 0.001) and ferritin (ρ = 0.234, *p* < 0.001), and with cellular injury as indicated by LDH (ρ = 0.334, *p* < 0.001). A pattern of immune dysregulation was evident, with severity showing a strong positive correlation with NLR (ρ = 0.299, *p* < 0.001) and neutrophil count (ρ = 0.181, *p* < 0.001), and a significant negative correlation with absolute lymphocyte count (ρ = −0.267, *p* < 0.001). Moderate correlations were observed with hypercoagulability markers (D-dimer: ρ = 0.132, *p* = 0.007), hepatic involvement (aspartate transaminase (AST): ρ = 0.146, *p* < 0.001) and established demographic risk factors (age: ρ = 0.133, *p* = 0.001; weight: ρ = 0.288, *p* < 0.001). Creatinine showed a significant but weak positive correlation (ρ = 0.098, *p* = 0.017) with disease severity. Among all comorbidities analyzed, only pulmonary disease and obesity were independently associated with a higher maximum disease severity score (Figure 2).

LOS was found to be associated with hypertension (ρ = 0.107; *p* = 0.009), dyslipidemia (ρ = 0.162; *p* < 0.001), renal (ρ = 0.123; *p* = 0.003), hematologic (ρ = 0.136; *p* < 0.001) and other (ρ = 0.135; *p* < 0.001) comorbidities; with plasma level of urea (ρ = 0.120; *p* = 0.004), ventilatory supplementation (ρ = 0.253; *p* < 0.001), remdesivir (ρ = 0.152; *p* < 0.001), enoxaparin (ρ = 0.136; *p* < 0.001), and enoxaparin regimen (ρ = 0.224; *p* < 0.001).

The results of the independent samples *t*-tests for all comorbidities are summarized visually in Figure 2. The forest plot displays the standardized mean difference (Cohen’s d) with 95% confidence intervals for each condition. As shown, only two comparisons yielded statistically significant effects: participants with pulmonary disease (Cohen’s d = −0.19, 95% CI [−0.37, −0.01]) and obesity (Cohen’s d = −0.22, 95% CI [−0.39, −0.05]) scored higher on the ordinal scale than their counterparts without these conditions.

### 3.4. Regressions

**Figure 3:**
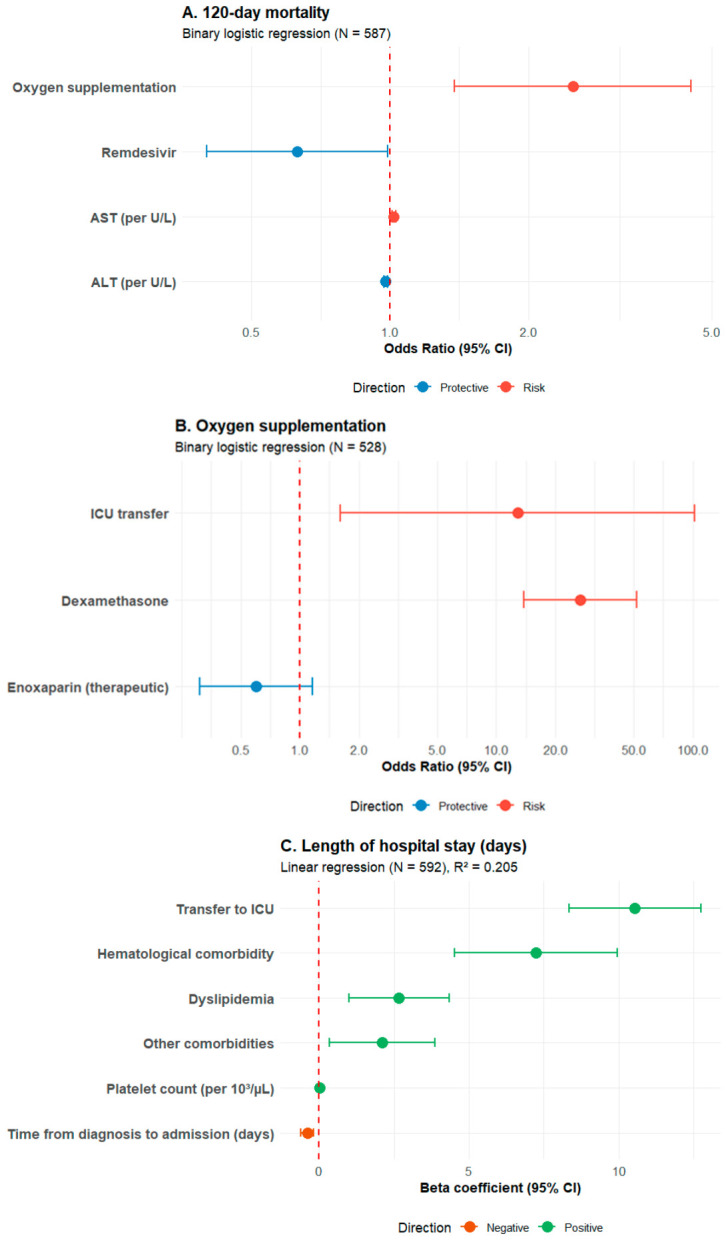
Forest plots of multivariable predictors. (**A**) Predictors of 120-day mortality (binary logistic regression, *N* = 587). (**B**) Predictors of oxygen supplementation (binary logistic regression, *N* = 528). (**C**) Predictors of length of hospital stay (linear regression, *N* = 592). Squares represent point estimates (odds ratio for (**A**,**B**); beta coefficient for (**C**)); error bars represent 95% confidence intervals. The red dashed line indicates a null effect (OR = 1 for (**A**,**B**); beta = 0 for (**C**)). Colors indicate effect direction: red = risk/increased outcome; blue = protective/decreased outcome; green = positive association; orange = negative association.

Multivariable logistic regression (Table 3) identified oxygen supplementation as the strongest independent predictor of 120-day all-cause mortality (OR = 2.50, 95% CI: 1.38–4.51, *p* = 0.002). Remdesivir administration was associated with reduced mortality (OR = 0.63, 95% CI: 0.40–0.99, *p* = 0.045). Higher AST levels independently predicted increased mortality (OR per U/L = 1.02, 95% CI: 1.01–1.03, *p* < 0.001), while higher ALT levels were associated with decreased mortality (OR per U/L = 0.98, 95% CI: 0.97–0.99, *p* = 0.001). The model demonstrated excellent calibration (Hosmer-Lemeshow *p* = 0.928) with acceptable explanatory power (Nagelkerke R^2^ = 0.080). Multivariable logistic regression (Table 4) identified dexamethasone administration as the strongest predictor of oxygen requirement (OR = 26.79, 95% CI: 13.84–51.86, *p* < 0.001), reflecting confounding by indication rather than a causal effect. ICU transfer was also independently associated with oxygen need (OR = 12.86, 95% CI: 1.61–102.60, *p* = 0.016). Enoxaparin dosing regimen was not significantly associated (*p* = 0.129). The model demonstrated excellent fit (Nagelkerke R^2^ = 0.485; Hosmer-Lemeshow *p* = 0.903). Multivariable linear regression (Table 5) showed that ICU transfer (B = 10.55 days, 95% CI: 8.36–12.75, β = 0.349, *p* < 0.001) and hematological comorbidities (B = 7.24 days, 95% CI: 4.52–9.95, β = 0.194, *p* < 0.001) were the strongest predictors of prolonged LOS. Dyslipidemia, other comorbidities, and higher platelet count were also associated with longer stays, while longer time from diagnosis to admission was associated with shorter stays. The model explained 20.5% of the variance in length of stay (adjusted R^2^ = 0.197, *p* < 0.001). The models are visualized in Figure 3.

### 3.5. Moderation Analysis

A positive correlation was found between LOS and oxygen supplementation invasiveness (ρ = 0.308; *p* < 0.001). A linear regression showed a significant moderating effect of ICU transfer status on this relationship (R^2^ = 0.132, F (3, 590) = 29.83, *p* < 0.001). The interaction was significant (B = 2.68, t = 3.67, *p* = 0.003), indicating the positive relationship between oxygen supplementation and LOS was stronger for patients transferred to the ICU.

## 4. Discussion

This study provides a comprehensive analysis of risk factors associated with acute severity, ICU admission, and post-discharge mortality in a cohort of hospitalized COVID-19 patients. Our findings reveal a clear dissociation between predictors of in-hospital outcomes and those associated with late mortality, underscoring the need for a phase-specific approach to risk stratification and patient management.

The overall mortality rate in our cohort was 22.5%, with more than one-third of deaths occurring after hospital discharge. This highlights that survival to discharge does not equate to recovery, as vulnerability persists beyond the acute phase, particularly in patients with malignancy, underscoring the importance of post-acute surveillance. ICU admission was strongly associated with obesity and male sex, consistent with previous reports linking these factors to early respiratory deterioration [5,6,7]. Previous studies have demonstrated that advanced age, male sex, and obesity are consistently linked to worse in-hospital outcomes across diverse healthcare settings [5,6,7]. In contrast, post-discharge mortality was independently associated with prolonged hospital stay and the presence of malignancy, but not with ICU admission. These findings suggest that while obesity and male sex are critical for predicting acute deterioration, malignancy is a key determinant of late adverse outcomes, regardless of ICU care.

Two mortality phases were identified, each associated with distinct patient characteristics. During the acute phase, obesity and male sex were associated with ICU admission, whereas late post-discharge mortality was specifically associated with malignancy, independent of ICU admission status. Length of stay further delineated these patterns: patients who died during hospitalization had the shortest stay (9.9 days), consistent with rapid clinical deterioration, whereas patients who died post-discharge had markedly prolonged hospitalization (18.4 days), suggesting that length of stay may have prognostic value beyond the acute phase. From a clinical perspective, this dissociation highlights the need to distinguish predictors of acute respiratory failure from predictors of late adverse outcomes when planning inpatient care and post-discharge follow-up.

The substantial proportion of deaths occurring after hospital discharge (37.3% of all deaths) underscores an important gap in the current understanding of COVID-19 outcomes. A longitudinal study has demonstrated persistently elevated mortality risk among hospitalized COVID-19 patients extending into the third year after infection [24], suggesting that the late mortality observed in our cohort likely represents the early phase of a prolonged vulnerability trajectory.

As expected, the laboratory profiles differentiating survivors from non-survivors reflected a systemic inflammatory and thromboinflammatory phenotype, characterized by elevated neutrophil counts, neutrophil to lymphocyte ratio (NLR), C-reactive protein (CRP), D-dimer, lactate dehydrogenase (LDH), and markers of renal dysfunction in non-survivors [15,16,17,18,25]. These biomarkers have practical utility, as CRP and D-dimer are routinely available in most hospital settings, including resource-limited environments [17,18]. The strong correlation between the WHO ordinal scale and mortality supports its continued use as a standardized prognostic tool [23,26]. Its strongest correlates, markers of inflammation (CRP, ferritin) and cellular injury (LDH), reflect the immune dysregulation that characterizes progression to organ failure [27]. The associations with elevated D-dimer and aspartate transaminase (AST) further link higher severity scores to thromboinflammatory state and hepatic involvement [5,17,28], while the weaker association with creatinine plasma levels suggests that renal dysfunction may not be a primary driver of the peak clinical severity. This biomarker specificity indicates that the WHO ordinal scale predominantly mirrors the acute hyperinflammatory and thromboinflammatory burden of hospitalized COVID-19 patients.

In line with the findings of Wajdowicz et al. (2026) [29], the need for oxygen supplementation (ventilatory support) was the strongest independent predictor of mortality in multivariable analysis. Hypoxemia therefore represents a key clinical signal of impending respiratory failure and overall disease burden, consistent with previous reports linking oxygen requirements to disease severity in hospitalized COVID-19 patients [5,28]. This observation aligns with the established inflammatory and thromboinflammatory pathophysiology of severe SARS-CoV-2 infection, characterized by endothelial injury, coagulation abnormalities, and multi-organ involvement [15,16,17].

Although cardiac, renal, and oncological comorbidities were associated with mortality in bivariate analyses, only pulmonary conditions and obesity independently predicted maximum disease severity. These findings suggest that these conditions may accelerate early pathophysiological processes, potentially through direct respiratory compromise [30] and metabolic-inflammatory dysregulation [31]. Indeed, obesity, an inflammatory disease [32], was associated with a more than two-fold increase in the odds of ICU admission, according to previous works linking obesity to hyperinflammatory responses in COVID-19 [31,33]. Clinically, this finding supports prioritizing early respiratory monitoring and timely escalation of care in hospitalized patients with obesity. The absence of independent associations for hypertension and diabetes with disease severity may reflect the widespread pharmacological management of these conditions [34].

In contrast, malignancy emerged as a key determinant of late post-discharge mortality, independent of ICU admission. Previous studies from Northern Portugal have reported increased mortality among cancer patients with SARS-CoV-2 infection [35], while pandemic-related disruptions in cancer screening and more advanced disease presentation have also been documented [36]. A recent Portuguese ICU study across six pandemic waves identified hematologic malignancy as a predictor of late ICU mortality [37] but did not capture deaths occurring after discharge. While Santos et al. [38] demonstrated that pre-ICU comorbidities collectively predict post-ICU mortality, our findings refine this observation by identifying malignancy as a specific predictor of post-discharge death. Several interconnected mechanisms may explain this vulnerability: baseline immunocompromise impairing viral clearance [35]; synergism between COVID-19 hyperinflammation and malignancy-related hypercoagulability [16,17]; pandemic-related disruptions to cancer care allowing disease progression [36]; and persistent ‘long COVID’ symptoms debilitating recovering patients [13]. By disaggregating comorbidities according to their role across the disease trajectory, our study addresses the need for targeted risk stratification and responds to calls for focused research on post-discharge outcomes [38]. Disentangling these mechanisms requires dedicated prospective studies with detailed cause-of-death adjudication.

Treatment effects in this observational cohort must be interpreted in the context of confounding by indication [39]. After adjustment for disease severity and biochemical profile, remdesivir was associated with improved survival, despite being preferentially administered to sicker patients. Enoxaparin use demonstrated a complex pattern consistent with severity-driven treatment allocation. The observed survival advantage associated with symptomatic presentation likely reflects diagnostic pathways during the study period, whereby asymptomatic infections were often detected through universal inpatient screening, identifying inherently more vulnerable patients with probable nosocomial infection [40].

Taken together, our findings demonstrate that COVID-19 mortality risk evolves across distinct temporal phases, with inflammatory and respiratory mechanisms predominating during hospitalization, while comorbidity-related vulnerability drives late post-discharge outcomes. This has important implications for clinical care and pandemic preparedness, as risk stratification models should differentiate predictors of acute deterioration from predictors of post-discharge mortality. Hospital-based surveillance alone may substantially underestimate COVID-19-related mortality, and preparedness strategies should incorporate structured, risk-based post-discharge monitoring, particularly for patients with malignancy and other conditions conferring sustained vulnerability. These findings may be especially informative in settings with low vaccination coverage or limited access to advanced therapeutics, where intrinsic determinants of disease severity and mortality remain clinically relevant.

### Limitations and Future Directions

This study has several strengths, including longitudinal follow-up through 120 days after discharge, comprehensive clinical and laboratory characterization, and the use of a pre-vaccine cohort providing a baseline understanding of disease determinants unmodified by vaccine-induced immunity. Limitations include the single-center observational design, which may limit generalizability to other healthcare settings with different patient demographics, resources, and practice patterns. The study period (September–November 2020) preceded widespread vaccination and the emergence of Variants of Concern [41]; therefore, the findings reflect the intrinsic pathogenicity of early SARS-CoV-2 lineages rather than contemporary disease patterns shaped by vaccine-induced immunity, prior infection, or altered viral virulence. Treatment protocols have also evolved substantially since 2020, with broader use of immunomodulators and antiviral agents, which may modify the risk factor profiles we observed. The use of the maximum WHO ordinal scale score precludes analysis of baseline severity. In addition, late deaths after discharge may reflect progression of underlying comorbidities rather than direct effects of acute infection. Detailed cause-of-death classification after hospital discharge was not uniformly available for all patients. Consequently, we cannot definitively determine whether late deaths in the malignancy subgroup were primarily attributable to COVID-19 sequelae, underlying cancer progression, or treatment-related disruptions. Unfortunately, we had no systematic data on arterial blood gas (ABG) parameters, as ABG sampling was performed at clinical discretion rather than systematically. These parameters [e.g., PaO_2_/FiO_2_ ratio and alveolar-arterial oxygen gradient (A-a)] are known predictors of severe COVID-19 pneumonia [42], and their absence may limit the granularity of our respiratory severity assessment. Moreover, smoking status data were unavailable and consequently, we could not adjust the multivariable models for this variable. Because smoking is a recognized risk factor for severe respiratory infections and cardiovascular diseases, residual confounding may have influenced the estimated effects of comorbidities on mortality.

While SINAVE data confirmed no prior infections before enrolment and showed very low reinfection rates (0.35%) during the study period, we cannot exclude undetected, asymptomatic, or unreported reinfections to SINAVE. Also, treatment allocation was not randomized, and residual confounding by unmeasured factors such as frailty and functional status cannot be excluded and should be considered when interpreting both in-hospital and post-discharge outcomes. Specifically, the Clinical Frailty Scale, was not systematically available in our electronic health records during the study period.

Finally, a limitation of our study is the inability to perform time-to-event analysis for post-discharge mortality since the exact date of death was not available for patients who died after hospital discharge. Consequently, we could not calculate the precise time from discharge to death, precluding Cox proportional hazards regression. Our primary outcome is therefore 120-day all-cause mortality, which captures the full mortality burden following hospitalization. Future studies with granular timing data should validate our phase-specific risk findings using survival analysis methods.

Future studies should validate these phase-specific risk profiles in multicenter and contemporary cohorts, including vaccinated patients and infections with emerging SARS-CoV-2 variants. Prospective validation studies should incorporate standardized arterial blood gas assessment, including PaO_2_/FiO_2_ ratio and A-a gradient, and data on smoking status, to enable more precise characterization of hypoxemia severity and improve early risk stratification. Longer post-discharge follow-up is needed to clarify the mechanisms and duration of post-discharge mortality, particularly among patients with malignancy. Integrating measures of frailty, functional status, and post-acute biomarkers may further refine post-discharge risk prediction. Interventional studies are warranted to determine whether targeted, risk-based post-discharge follow-up can reduce post-discharge mortality.

## 5. Conclusions

This study shows that risk factors associated with acute COVID-19 severity and ICU admission differ from those associated with mortality after hospital discharge. Obesity and male sex were linked to acute deterioration requiring ICU care, whereas malignancy was associated with late post-discharge mortality, independent of ICU admission. With more than one-third of deaths occurring after discharge, these findings support a phase-specific approach to risk stratification that extends beyond hospitalization. Differentiating inpatient and post-discharge risk profiles may improve continuity of care and reduce late mortality in hospitalized patients with SARS-CoV-2 infection.

## Figures and Tables

**Table 1:** Differences in biochemical markers and comorbidities between outcome groups (alive vs. dead).

	Group	*N*	*M*	*SD*	*t*	*df*	*p*		Cohen’s *d*	
Neutrophils ×10^3^/µL	Alive	461	5.39	3.08	−2.189	178.301	**0.030**		−0.249	
	Dead	132	6.21	3.98						
NLR	Alive	461	5.96	4.79	−2.910	161.757	**0.004**		−0.367	
	Dead	132	8.00	7.62						
Hemoglobin (g/dL)	Alive	461	12.92	1.90	2.294	187.495	**0.023**		0.249	
	Dead	132	12.42	2.26						
Urea (mg/dL)	Alive	461	51.10	38.18	−4.139	180.664	**<0.001**		−0.249	
	Dead	132	69.95	48.18						
Glucose (mg/dL)	Alive	456	138.02	62.00	−2.067	584	**0.039**		−0.206	
	Dead	130	151.65	79.70						
ALP (U/L)	Alive	457	74.27	37.97	−3.242	170.409	**0.001**		−0.382	
	Dead	130	90.07	51.75						
LDH (U/L)	Alive	452	319.84	122.62	−4.185	167.695	**<0.001**		−0490	
	Dead	126	401.75	172.40						
CRP (mg/L)	Alive	461	86.76	69.66	−2.392	591	**0.017**		−0.236	
	Dead	132	109.37	63.41						
D-dimer (ng/mL)	Alive	334	864.19	883.57	−2.736	103.627	**0.007**		−0.426	
	Dead	86	1292.03	1378.93						
Comorbidity			Without Comorbidity *n* (%)	With Comorbidity *n* (%)	*N*		*X* ^2^	*df*	*p*-value	Φ
Cardiac	Alive		292 (63.3%)	169 (36.7%)	461		16.004	1	**<0.001**	0.164
	Dead		59 (44.0%)	75 (56.0%)	134					
Renal	Alive		372 (80.7%)	89 (19.3%)	461		7.752	1	**0.009**	0.114
	Dead		93 (69.4%)	41 (30.6%)	134					
Oncological	Alive		426 (92.4%)	35 (7.6%)	461		14.018	1	**<0.001**	0.153
	Dead		109 (81.3%)	25 (18.7%)	134					
Other	Alive		169 (36.7%)	292 (63.3%)	461		4.410	1	**0.036**	0.086
	Dead		36 (26.9%)	98 (73.1%)	134					

Legend—*N* = frequencies; *M* = mean; *SD* = standard deviation; *t* = *t*-test; *df* = degrees of freedom; *p* = *p*-value; Cohen’s *d* = size effect; ALP = alkaline phosphatase; LDH = lactate dehydrogenase; CRP = C-reactive protein; NLR = neutrophil-to-lymphocyte ratio. *X*^2^ = Chi-Squared test value; *df* = degrees of freedom; Φ = Phi coefficient (effect size). Significant *p*-values are in bold.

**Table 2:** Differences in biochemical markers and comorbidities concerning the oxygen supplementation outcome.

	*O*_2_ *Suppl.*	*N*	*M*	*SD*	*t*	*df*	*p*	Cohen’s *d*
Hemoglobin (g/dL)	No	129	12.23	2.14	−3.707	591	<0.001	−0.37
	Yes	464	12.96	1.93				
Neutrophils ×10^3^/µL	No	129	4.59	2.80	−3.858	591	<0.001	−0.38
	Yes	464	5.84	3.40				
NLR	No	129	3.94	2.79	−8.534	456,985	<0.001	−0.58
	Yes	464	7.10	5.98				
Glucose (mg/dL)	No	125	124.56	50.82	−3.755	263,439	<0.001	−0.32
	Yes	461	145.51	69.51				
ALP (U/L)	No	125	84.86	37.47	2.14	585	0.033	0.22
	Yes	462	75.85	42.84				
LDH (U/L)	No	124	273.54	94.48	−7.183	284,221	<0.001	−0.59
	Yes	454	350.40	138.92				
CRP (mg/L)	No	129	46.99	50.99	−10.048	273,598	<0.001	−0.84
	Yes	464	102.51	69.38				
Ferritin (ng/mL)	No	58	972.12	1062.51	−2.432	378	0.015	−0.35
	Yes	322	1392.73	1237.16				
Comorbidity	O_2_ Suppl.	Without Comorbidity *n* (%)	With Comorbidity *n* (%)	Total	*X* ^2^	*df*	*p*-value	*Φ*
Pulmonary	No	107 (82.3%)	23 (17.7%)	130	9.384	1	0.002	0.126
	Yes	319 (68.6%)	146 (31.4%)	465				
Obesity	No	100 (76.9%)	30 (23.1%)	130	7.327	1	0.007	0.111
	Yes	299 (64.3%)	166 (35.7%)	465				
Diabetes	No	95 (73.1%)	35 (26.9%)	130	4.002	1	0.045	0.082
	Yes	296 (63.7%)	169 (36.3%)	465				

*Legend. N* = frequencies; *M* = mean; *SD* = standard deviation; *t* = *t*-test; *df* = degrees of freedom; *p* = *p*-value; Cohen’s *d* = size effect; ALP = alkaline phosphatase; LDH = lactate dehydrogenase; CRP = C-reactive protein; NLR= neutrophil-to-lymphocyte ratio. *X*^2^ = Chi-Squared test value; *df* = degrees of freedom; Φ = Phi coefficient (effect size).

**Table 3:** Multivariable logistic regression for 120-day all-cause mortality (*N* = 587).

Variable	B	SE	Wald	*p*-Value	OR	95% CI
Oxygen supplementation	0.915	0.301	9.216	0.002	2.50	1.38–4.51
Remdesivir	−0.457	0.228	4.009	0.045	0.63	0.40–0.99
AST (per U/L)	0.020	0.005	17.596	<0.001	1.02	1.01–1.03
ALT (per U/L)	−0.016	0.005	10.791	0.001	0.98	0.97–0.99
Constant	−2.256	0.304	54.994	<0.001	0.11	–

Legend. Model fit: χ^2^ (4) = 31.29, *p* < 0.001; Nagelkerke R^2^ = 0.080; Hosmer-Lemeshow χ^2^ (8) = 3.10, *p* = 0.928; Classification accuracy: 78.7% overall; sensitivity 6.2%, specificity 99.1%. ALT = Alanine aminotransferase; AST = Aspartate aminotransferase.

**Table 4:** Multivariable logistic regression for oxygen supplementation (*N* = 528).

Variable	B	SE	Wald	*p*-Value	OR	95% CI
ICU transfer	2.554	1.060	5.803	0.016	12.86	1.61–102.60
Dexamethasone	3.288	0.337	95.364	<0.001	26.79	13.84–51.86
Enoxaparin dose (therapeutic vs. prophylactic)	−0.517	0.341	2.303	0.129	0.60	0.31–1.16
Constant	0.454	0.444	1.044	0.307	1.57	–

Legend. Model fit: χ^2^ (3) = 187.15, *p* < 0.001; Nagelkerke R^2^ = 0.485; Hosmer-Lemeshow χ^2^ (4) = 1.05, *p* = 0.903; Classification accuracy: 84.1% overall; sensitivity 83.5%, specificity 86.6%. ICU = intensive care unit.

**Table 5:** Linear regression for length of hospital stay (days) (*N* = 592).

Variable	B	95% CI	β	*p*-Value
Transfer to ICU	10.55	8.36–12.75	0.349	<0.001
Hematological comorbidity	7.24	4.52–9.95	0.194	<0.001
Dyslipidemia	2.67	1.00–4.34	0.116	0.002
Other comorbidities	2.11	0.35–3.87	0.087	0.019
Platelet count (per 10^3^/µL)	0.013	0.004–0.022	0.106	0.004
Time from diagnosis to admission (days)	−0.392	−0.612–−0.172	−0.130	<0.001
Constant	5.21	2.59–7.82	–	<0.001

Legend: Model fit: F(6, 585) = 25.11, *p* < 0.001; R^2^ = 0.205; adjusted R^2^ = 0.197; B = unstandardized coefficient; 95% CI = confidence interval; β = standardized coefficient; ICU = intensive care unit.

## Data Availability

The original contributions presented in this study are included in the article/Supplementary Materials. Further inquiries can be directed to the corresponding author.

## Supplementary Materials

The following supporting information can be downloaded at: https://www.mdpi.com/article/10.3390/medsci14020255/s1, S1 File. STROBE Checklist. Completed STROBE checklist for cohort studies. S2 File. Tables S1–S5 contain additional analyses and Figure S1 represents the flow diagram of study participants.
