# Supplementary material for: ICU Admission and Post-Discharge Mortality in COVID-19: Different Risk Factors Across Clinical Phases
Source: Med Sci (Basel). 2026 May 14;14(2):255. doi: 10.3390/medsci14020255 (PMC13214800; doi:10.3390/medsci14020255)
Supplement: Supplementary file 1 [file medsci-14-00255-s001.zip › STROBE checklist Final S1 File.pdf]

STROBE Statement – Checklist for cohort studies

Item	Recommendation	Section/Paragraph	Line Numbers
**Title**	ICU Admission and Post-Discharge Mortality in COVID-19: Different Risk Factors across Clinical Phases	Title	L2
**Authors**	Fernanda Leite MD MSc PhD; André Santos Silva MD; Sara Ferreira MD; Carina Brito MD; Ângela Leite PhD	Authors	L4
**1a**	Indicate the study's design with a commonly used term in the title or the abstract	Title; Abstract	Title: L2; Abstract: L13-L37
**1b**	Provide in the abstract an informative and balanced summary of what was done and what was found	Abstract	L13-L37
**2**	Explain the scientific background and rationale for the investigation being reported	Introduction paragraphs 1-6	L38-82
**3**	State specific objectives, including any prespecified hypotheses	Introduction paragraph 7	L72-77
**4**	Present key elements of study design early in the paper	Methods 2.1	L84-108
**5**	Describe the setting, locations, and relevant dates, including periods of recruitment, exposure, follow-up, and data collection	Methods 2.1	L84-108
**6a**	Give the eligibility criteria, and the sources and methods of selection of participants	Methods 2.1	L84-108
**6b**	For matched studies, matching criteria and number of exposed and unexposed	N/A	N/A
**7**	Clearly define all outcomes, exposures, predictors, potential confounders, and effect modifiers. Give diagnostic criteria, if applicable	Methods 2.2; 2.3; 2.4	L109-134
**8**	For each variable of interest, give sources of data and details of methods of assessment (measurement)	Methods 2.2; 2.3; 2.4;	L109-134
**9**	Describe any efforts to address potential sources of bias	Methods 2.5	L135-175
**10**	Explain how the study size was arrived at	Methods 2.1	L84-108
**11**	Explain how quantitative variables were handled in the analysis. If applicable, describe which groupings were chosen and why	Methods 2.5	L135-175
**12a**	Describe all statistical methods, including those used to control for confounding	Methods 2.5	L135-175
**12b**	Describe any methods used to examine subgroups and interactions	Methods 2.5; Results 3.4-3.5	L135-175; L342-409
**12c**	Explain how missing data were addressed	Methods 2.5	L135-175

**12d**	If applicable, explain how loss to follow-up was addressed	Methods 2.4	L129-134
**12e**	Describe any sensitivity analyses	N/A (stated L31: "No additional sensitivity analyses were performed.")	N/A
**13a**	Report numbers of individuals at each stage of study	Results 3.1, figure S1	L177-200
**13b**	Give reasons for non-participation at each stage	N/A	N/A
**13c**	Consider use of a flow diagram	Supporting Information	Figure S1
**14a**	Give characteristics of study participants and information on exposures and potential confounders	Results; Table 1	
**14b**	Indicate number of participants with missing data for each variable of interest	Methods 2.5; Tables	Tables
**15**	Indicate number of outcome events or summary measures	Results 3.1	L177- 200
**16a**	Give unadjusted estimates and confounder-adjusted estimates with 95% confidence intervals	Results 3.2-3.4; Tables 1-2; Supplementary Tables 4-8	L225-363; Table 1; Table 2: Regressions: Tables3,4,5
**16b**	Report category boundaries when continuous variables were categorized	Methods 2.2; 2.3	L109-128
**16c**	If relevant, consider translating estimates of relative risk into absolute risk	N/A - absolute risks are presented in descriptive tables (Table 1, Supplementary Table 1) allowing readers to calculate absolute differences directly. For example, obesity: 47.6% ICU admission with obesity vs. 30.0% without (absolute difference 17.6%; OR=2.12).	Supplementary Material, Tables
**17**	Report other analyses done: e.g., analyses of subgroups and interactions	Results 3.5	L403-409
**18**	Summarize key results with reference to study objectives	Discussion paragraph 1	L411-416
**19**	Discuss limitations of the study, considering sources of potential bias or imprecision	Discussion paragraph limitations section	L515-570
**20**	Give a cautious overall interpretation of results considering objectives, limitations, and other relevant evidence	Discussion (all paragraphs)	L411-579

**21**	Discuss the generalizability (external validity) of the study results	Limitations section	L523-525
**22**	Give the source of funding and the role of the funders	Funding section	N/A
