# Supplementary material for: ICU Admission and Post-Discharge Mortality in COVID-19: Different Risk Factors Across Clinical Phases
Source: Med Sci (Basel). 2026 May 14;14(2):255. doi: 10.3390/medsci14020255 (PMC13214800; doi:10.3390/medsci14020255)
Supplement: Supplementary file 1 [file medsci-14-00255-s001.zip › Supplementary Material S2 File_13_05_2026.pdf]

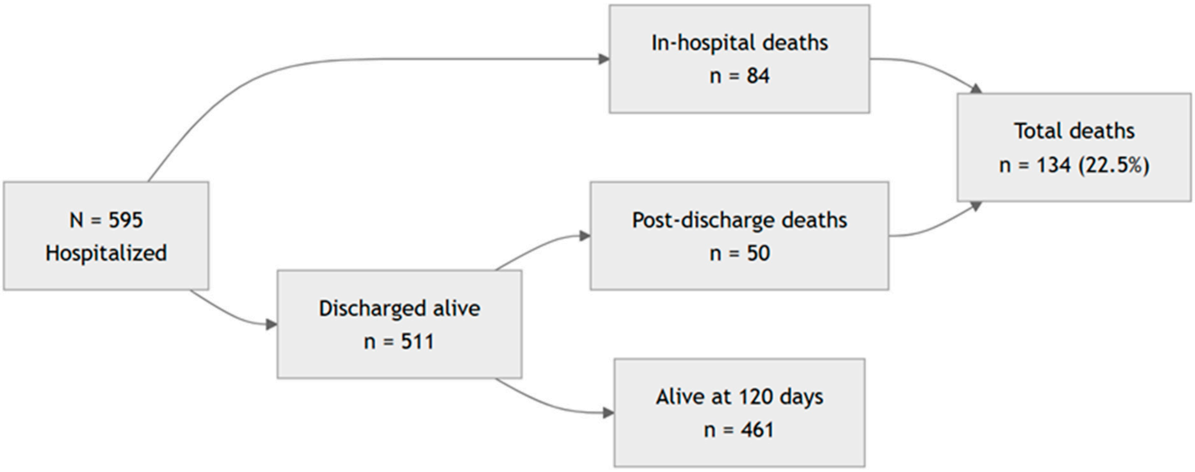

Figure S1. Flow diagram of study participants. A total of 595 adults hospitalized with PCR-confirmed COVID-19 between September 1 and November 30, 2020, were included. In-hospital mortality was 14.1% (n = 84). Among 511 patients discharged alive, 50 additional deaths occurred by 120-day follow-up (March 2021), representing 37.3% of all deaths. Total mortality at 120-day follow-up was 22.5% (n = 134). Patients still hospitalized at data extraction were excluded from mortality and length of stay calculations per Methods 2.4.

Table S1. Comparison of patient characteristics by ICU admission status

Characteristic	Category	Non-ICU (*N* = 490)	ICU (*N* = 105)	*OR*	95% *CI*	χ^2	*p*-value
Mortality				3.21	(2.05–5.02)	27.455	**<0.001**
	Alive	400 (81.6)	61 (58.1)	1.00	Reference		
	Dead	90 (18.4)	44 (41.9)	3.21	(2.05–5.02)		
Sex				1.78	(1.14–2.78)	6.671	**0.010**
	Female	231 (47.1)	35 (33.3)	1.00	Reference		
	Male	259 (52.9)	70 (66.7)	1.78	(1.14–2.78)		
Pulmonary disease				0.90	(0.56–1.44)	0.189	0.664
	Without	349 (71.2)	77 (73.3)	1.00	Reference		
	With	141 (28.8)	28 (26.7)	0.90	(0.56–1.44)		
Cardiac disease				0.74	(0.48–1.15)	1.755	0.185
	Without	283 (57.8)	68 (64.8)	1.00	Reference		
	With	207 (42.2)	37 (35.2)	0.74	(0.48–1.15)		
Hypertension				1.24	(0.80–1.93)	0.935	0.334
	Without	188 (38.4)	35 (33.3)	1.00	Reference		
	With	302 (61.6)	70 (66.7)	1.24	(0.80–1.93)		
Dyslipidemia				0.90	(0.59–1.38)	0.219	0.640
	Without	249 (50.8)	56 (53.3)	1.00	Reference		
	With	241 (49.2)	49 (46.7)	0.90	(0.59–1.38)		
Obesity				2.12	(1.39–3.23)	12.120	**<0.001**
	Without	343 (70.0)	55 (52.4)	1.00	Reference		
	With	147 (30.0)	50 (47.6)	2.12	(1.39–3.23)		
Diabetes				1.20	(0.78–1.85)	0.680	0.410
	Without	324 (66.1)	65 (61.9)	1.00	Reference		
	With	166 (33.9)	40 (38.1)	1.20	(0.78–1.85)		
Renal disease				1.08	(0.65–1.78)	0.076	0.783
	Without	384 (78.4)	81 (77.1)	1.00	Reference		
	With	106 (21.6)	24 (22.9)	1.08	(0.65–1.78)		
Hematological disease				0.55	(0.24–1.25)	2.071	0.150
	Without	434 (88.6)	98 (93.3)	1.00	Reference		
	With	56 (11.4)	7 (6.7)	0.55	(0.24–1.25)		
Oncological disease				0.70	(0.32–1.51)	0.854	0.355
	Without	438 (89.4)	97 (92.4)	1.00	Reference		
	With	52 (10.6)	8 (7.6)	0.70	(0.32–1.51)		
Other comorbidities				0.79	(0.51–1.21)	1.191	0.275
	Without	164 (33.5)	41 (39.0)	1.00	Reference		
	With	326 (66.5)	64 (61.0)	0.79	(0.51–1.21)		

Legend - Data are presented as *N* (%); ICU = intensive care unit; *OR* = odds ratio; *CI* = confidence interval; Percentages are column percentages. χ^2: Pearson's chi-square test. Bold p-values indicate statistical significance ($p < 0.05$).

Table S2. Differences Between Remdesivir and Non-Remdesivir Groups

Variable	*Group*	*N*	*M*	*SD*	*t*	*df*	*p-value*	*Cohen's d*
**Age (years)**	Remdesivir	202	71.45	15.51	2.234	593	**0.026**	0.193
	No-Remdesivir	393	68.50	14.61				
**NLR**	Remdesivir	202	7.21	5.76	-2.508	591	**0.012**	-0.217
	No-Remdesivir	391	6.00	5.48				
**Hemoglobin (g/dL)**	Remdesivir	202	13.40	1.75	5.603	466.11	**<0.001**	0.462
	No-Remdesivir	391	12.50	2.05				
**Platelets (x10³/µL)**	Remdesivir	202	199.45	94.36	2.482	591	**0.013**	0.215
	No-Remdesivir	391	219.86	95.18				
**Urea (mg/dL)**	Remdesivir	202	45.61	25.82	4.931	588.29	**<0.001**	-0.360
	No-Remdesivir	391	60.29	46.64				
**Creatinine (mg/dL)**	Remdesivir	202	1.03	0.40	5.150	512.47	**<0.001**	-0.340
	No-Remdesivir	391	1.39	1.30				
**Bilirubin (mg/dL)**	Remdesivir	202	0.478	0.225	2.131	507	**0.034**	-0.142
	No-Remdesivir	385	0.563	0.718				
**ALP (U/L)**	Remdesivir	202	70.73	41.64	2.970	585	**0.003**	0.258
	No-Remdesivir	385	81.46	41.60				
**LDH (U/L)**	Remdesivir	201	362.43	140.10	-3.768	576	**<0.001**	-0.329
	No-Remdesivir	377	318.70	128.84				
**CRP (mg/L)**	Remdesivir	202	108.94	66.17	-4.735	591	**<0.001**	-0.410
	No-Remdesivir	391	80.87	69.57				
**Ferritin (ng/mL)**	Remdesivir	161	1489.81	1246.89	-2.220	378	**0.027**	0.231
	No-Remdesivir	219	1209.97	1189.27				

Legend - *N* = sample size; *M* = Mean; *SD* = Standard Deviation; *t* = t-test value; *df* = degrees of freedom; Cohen's *d* = effect size. ALP = alkaline phosphatase; LDH = lactate dehydrogenase; CRP = C-reactive protein; NLR = neutrophil-to-lymphocyte ratio. Significant *p*-values are in bold.

Table S3. Differences Between Asymptomatic and Symptomatic Patients

Variable	*Group*	*N*	*M*	*SD*	*t*	*p-value*	*Cohen's d*
**AST (U/L)**	Asymptomatic	158	39.76	22.39	-2.26	**0.024**	-0.160
	Symptomatic	429	46.02	43.90			
**Total Bilirubin (mg/dL)**	Asymptomatic	155	0.56	0.44	2.00	**0.047**	0.226
	Symptomatic	430	0.49	0.29			
**CRP (mg/L)**	Asymptomatic	162	78.43	65.48	-2.58	**0.010**	-0.240
	Symptomatic	431	94.94	70.71			
Variable	*Group*	*N*	*No n (%)*	*Yes n (%)*	*χ^2(df)*	*p-value*	*Φ*
**Oxygen Supplementation**	Asymptomatic	162	72 (44.4%)	90 (55.6%)	66.563 (1)	**<0.001**	0.334
	Symptomatic	433	58 (13.4%)	375 (86.6%)			
**Dexamethasone**	Asymptomatic	162	77 (47.5%)	85 (52.5%)	14.596 (1)	**<0.001**	0.157
	Symptomatic	433	133 (30.7%)	300 (69.3%)			
**Enoxaparin**	Asymptomatic	162	23 (14.2%)	139 (85.8%)	4.154 (1)	**0.042**	0.084
	Symptomatic	433	37 (8.5%)	396 (91.5%)			

Legend - *M* = Mean; *SD* = Standard Deviation; *t* = t-test value; Cohen's *d* = effect size. AST = aspartate transaminase; CRP = C-reactive protein. N = sample size; % = percentage; χ^2 = Chi-Squared test value; *df* = degrees of freedom; *Φ* = Phi coefficient (effect size). Significant *p*-values are in bold.

Table S4. Significant Correlations between 120-Day All-Cause Mortality and clinical and laboratory variables

Variable Group		Correlation Coefficient (ρ)	p-value
**Comorbidities**	Cardiac Disease	0.164	<0.001
	Renal Disease	0.114	0.005
	Oncological Disease	0.153	<0.001
	Other Comorbidities	0.086	0.036
**Laboratory parameters**	NLR	0.102	0.013
	Lymphocyte Count	-0.093	0.023
	Creatinine	0.139	<0.001
	Glucose	0.108	0.009
	Urea	0.227	<0.001
	Aspartate Aminotransferase	0.107	0.009
	Alkaline Phosphatase	0.125	0.002
	Lactate Dehydrogenase	0.178	<0.001
	C-reactive Protein	0.120	0.003
	D-dimer	0.171	<0.001
**Clinical characteristics**	Oxygen Supplementation	0.360	<0.001
	Therapeutic Enoxaparin Regimen*	0.226	<0.001
	Days from Diagnosis to Admission	-0.120	0.003
	Enoxaparin Intake	-0.113	0.006

Legend - ρ = Spearman's rank correlation coefficient. *The positive correlation with the therapeutic enoxaparin and the negative correlation with enoxaparin intake suggest treatment was stratified by severity, with higher-risk more likely to receive therapeutic dosing.

Table S5. Univariable comparisons of clinical and laboratory variables according to 120-day mortality, oxygen requirement, and remdesivir administration.

Variable	Group	Mortality (Alive vs Dead)	Oxygen (without vs with)	Remdesivir (no vs yes)
**Neutrophils ($x10^3/\mu L$)**	Reference	5.39 ± 3.08	4.59 ± 2.80	5.52 ± 3.40
	Exposed	6.21 ± 3.98	5.84 ± 3.40	5.67 ± 3.16
	Test statistic	t(178.3) = -2.189	t(591) = -3.858	t(591) = -0.502
	p-value	**p = 0.030**	**p < 0.001**	p = 0.616
**NLR**	Reference	5.96 ± 4.79	3.94 ± 2.79	6.00 ± 5.48
	Exposed	8.00 ± 7.62	7.10 ± 5.98	7.21 ± 5.76
	Test statistic	t(161.8) = -2.910	t(456.9) = -8.534	t(591) = -2.508
	p-value	**p = 0.004**	**p < 0.001**	**p = 0.012**
**Hemoglobin (g/dL)**	Reference	12.92 ± 1.90	12.23 ± 2.14	12.50 ± 2.05
	Exposed	12.42 ± 2.26	12.96 ± 1.93	13.40 ± 1.75
	Test statistic	t(187.5) = 2.294	t(591) = -3.707	t(466.1) = 5.603
	p-value	**p = 0.023**	**p < 0.001**	**p < 0.001**
**Urea (mg/dL)**	Reference	51.10 ± 38.18	52.51 ± 41.09	60.29 ± 46.64
	Exposed	69.95 ± 48.18	56.07 ± 41.40	45.61 ± 25.82
	Test statistic	t(180.7) = -4.139	t(591) = -0.863	t(588.3) = 4.931
	p-value	**p < 0.001**	p = 0.388	**p < 0.001**
**Glucose (mg/dL)**	Reference	138.02 ± 62.00	124.56 ± 50.82	139.07 ± 65.56
	Exposed	151.65 ± 79.70	145.51 ± 69.51	144.81 ± 68.22
	Test statistic	t(584) = -2.067	t(263.4) = -3.755	t(584) = -0.992
	p-value	**p = 0.039**	**p < 0.001**	p = 0.322
**ALP (U/L)**	Reference	74.27 ± 37.97	84.86 ± 37.47	81.46 ± 41.60
	Exposed	90.07 ± 51.75	75.85 ± 42.84	70.73 ± 41.64
	Test statistic	t(170.4) = -3.242	t(585) = 2.140	t(585) = 2.970
	p-value	**p = 0.001**	**p = 0.033**	**p = 0.003**
**LDH (U/L)**	Reference	319.84 ± 122.62	273.54 ± 94.48	318.70 ± 128.84
	Exposed	401.75 ± 172.40	350.40 ± 138.92	362.43 ± 140.10
	Test statistic	t(167.7) = -4.185	t(284.2) = -7.183	t(576) = -3.768
	p-value	**p < 0.001**	**p < 0.001**	**p < 0.001**
**CRP (mg/L)**	Reference	86.76 ± 69.66	46.99 ± 50.99	80.87 ± 69.57
	Exposed	109.37 ± 63.41	102.51 ± 69.38	108.94 ± 66.17
	Test statistic	t(591) = -2.392	t(273.6) = -10.048	t(591) = -4.735
	p-value	**p = 0.017**	**p < 0.001**	**p < 0.001**
**D-dimer (ng/mL)**	Reference	864.19 ± 883.57	996.64 ± 1107.78	989.60 ± 1000.73
	Exposed	1292.03 ± 1378.93	944.47 ± 1003.88	895.10 ± 1043.51
	Test statistic	t(103.6) = -2.736	t(418) = 0.365	t(418) = 0.932
	p-value	**p = 0.007**	p = 0.716	p = 0.352
**Ferritin (ng/mL)**	Reference	1301.22 ± 1241.99	972.12 ± 1062.51	1209.97 ± 1189.27
	Exposed	1423.31 ± 1143.66	1392.73 ± 1237.16	1489.81 ± 1246.89
	Test statistic	t(378) = -0.812	t(378) = -2.432	t(378) = -2.220
	p-value	p = 0.417	**p = 0.015**	**p = 0.027**
**Platelets ($x10^3/\mu L$)**	Reference	216.89 ± 93.10	213.67 ± 98.51	219.86 ± 95.18
	Exposed	199.02 ± 101.83	212.70 ± 94.51	199.45 ± 94.36
	Test statistic	t(591) = 1.903	t(591) = 0.103	t(591) = 2.482
	p-value	p = 0.058	p = 0.918	**p = 0.013**
**Total Bilirubin (mg/dL)**	Reference	0.51 ± 0.57	0.69 ± 1.16	0.56 ± 0.72
	Exposed	0.61 ± 0.67	0.49 ± 0.29	0.48 ± 0.23
	Test statistic	t(585) = -1.563	t(128.1) = 1.923	t(507) = 2.131
	p-value	p = 0.119	p = 0.057	**p = 0.034**
	Reference	1.23 ± 1.10	1.25 ± 1.18	1.39 ± 1.30

**Creatinine (mg/dL)**	Exposed	1.40 ± 1.06	1.27 ± 1.07	1.03 ± 0.40
	Test statistic	t(591) = -1.613	t(591) = -0.212	t(512.5) = 5.150
	p-value	p = 0.107	p = 0.832	**p < 0.001**
**Cardiac disease (%)**	Reference	292 (63.3%)	74 (56.9%)	221 (56.2%)
	Exposed	75 (56.0%)	188 (40.4%)	72 (35.6%)
	Test statistic	$\chi^2(1) = 16.004$	$\chi^2(1) = 0.294$	$\chi^2(1) = 3.639$
	p-value	**p < 0.001**	p = 0.588	p = 0.056
**Renal disease (%)**	Reference	372 (80.7%)	95 (73.1%)	292 (74.3%)
	Exposed	41 (30.6%)	95 (20.4%)	29 (14.4%)
	Test statistic	$\chi^2(1) = 7.752$	$\chi^2(1) = 2.508$	$\chi^2(1) = 10.054$
	p-value	**p = 0.009**	p = 0.113	**p = 0.002**
**Oncological disease (%)**	Reference	426 (92.4%)	111 (85.4%)	348 (88.5%)
	Exposed	25 (18.7%)	41 (8.8%)	15 (7.4%)
	Test statistic	$\chi^2(1) = 14.018$	$\chi^2(1) = 3.767$	$\chi^2(1) = 2.383$
	p-value	**p < 0.001**	p = 0.052	p = 0.123
**Pulmonary disease (%)**	Reference	335 (78.6%)	107 (82.3%)	280 (71.2%)
	Exposed	43 (25.4%)	146 (31.4%)	56 (27.7%)
	Test statistic	$\chi^2(1) = 1.156$	$\chi^2(1) = 9.384$	$\chi^2(1) = 0.070$
	p-value	p = 0.282	**p = 0.002**	p = 0.792
**Obesity (%)**	Reference	312 (78.4%)	100 (76.9%)	268 (68.2%)
	Exposed	48 (24.4%)	166 (35.7%)	72 (35.6%)
	Test statistic	$\chi^2(1) = 0.574$	$\chi^2(1) = 7.327$	$\chi^2(1) = 0.887$
	p-value	p = 0.449	**p = 0.007**	p = 0.346
**Diabetes (%)**	Reference	309 (79.4%)	95 (73.1%)	258 (65.6%)
	Exposed	54 (26.2%)	169 (36.3%)	71 (35.1%)
	Test statistic	$\chi^2(1) = 2.462$	$\chi^2(1) = 4.002$	$\chi^2(1) = 0.037$
	p-value	p = 0.117	**p = 0.045**	p = 0.846

Legend: Data are presented as mean ± *SD* for continuous variables and n (%) for categorical variables. Reference groups: alive (mortality), without ventilatory treatment (oxygen), no remdesivir (remdesivir). Exposed groups: dead (mortality), with ventilatory treatment (oxygen), yes remdesivir (remdesivir). Statistics: *t (df)* = Student's t-test; χ^2 (df) = Chi-square test. Bold *p*-values indicate statistical significance ($p < 0.05$). NLR = neutrophil-to-lymphocyte ratio; ALP = alkaline phosphatase; LDH = lactate dehydrogenase; CRP = C-reactive protein.
